# Adaptive Curved Slicing for En Face Imaging in Optical Coherence Tomography

**DOI:** 10.3390/s25144329

**Published:** 2025-07-10

**Authors:** Mingxin Li, Phatham Loahavilai, Yueyang Liu, Xiaochen Li, Yang Li, Liqun Sun

**Affiliations:** 1 State Key Laboratory of Precision Measurement Technology and Instrument, Department of Precision Instrument, Tsinghua University, Beijing 100084, China; limx19@mails.tsinghua.edu.cn (M.L.); liugx23@mails.tsinghua.edu.cn (P.L.); liuyueya21@mails.tsinghua.edu.cn (Y.L.); lixiaochen@tsinghua.edu.cn (X.L.); liyang328@mail.sysu.edu.cn (Y.L.); 2 State Key Laboratory of Optoelectronic Materials and Technologies, School of Electronics and Information Technology, Sun Yat-sen University, Guangzhou 510275, China

**Keywords:** 3D metrology, optical coherence tomography, image processing, surface morphology fitting, functional sensing

## Abstract

Optical coherence tomography (OCT) employs light to acquire high-resolution 3D images and is widely applied in fields such as ophthalmology and forensic science. A popular technique for visualizing the top view (en face) is to slice it with flat horizontal plane or apply statistical functions along the depth axis. However, when the target appears as a thin layer, strong reflections from other layers can interfere with the target, rendering the flat-plane approach ineffective. We apply Otsu-based thresholding to extract the object’s foreground, then use least squares (with Tikhonov regularization) to fit a polynomial curve that describes the sample’s structural morphology. The surface is then used to obtain the latent fingerprint image and its residues at different depths from a translucent tape, which cannot be analyzed using conventional en face OCT due to strong reflection from the diffusive surface, achieving FSIM of 0.7020 compared to traditional en face of 0.6445. The method is also compatible with other signal processing techniques, as demonstrated by a thermal-printed label ink thickness measurement confirmed by a microscopic image. Our approach empowers OCT to observe targets embedded in samples with arbitrary postures and morphology, and can be easily adapted to various optical imaging technologies.

## 1. Introduction

Optical coherence tomography (OCT) is a powerful imaging technique that employs light to capture detailed, non-invasive images of subsurface structures. With superior axial resolution, OCT has revolutionized 3D imaging in various fields, including ophthalmology [1,2,3], dentistry [4], and industrial applications such as precision component testing [5]. Moreover, OCT has found significant use in forensic science, particularly in identifying counterfeits [6,7], automotive paint trace inspection [8], and analyzing fingerprints [9,10,11], due to its ability to reveal details obscured under a surface without damaging the evidence.

Standard OCT imaging retrieves a single spatial-axis intensity, which can be extended to form volumetric structural information by scanning in two additional orthogonal directions. In order to display the internal structure, cross-sectional images of the sample can be obtained by slicing along the depth direction of the OCT volume [12,13,14], while en face images are typically extracted by slicing horizontally at a specific depth [6] or applying statistical functions (such as average or maximum intensities) along the depth axis to realize a global projection [15].

Traditional en face image uses a flat horizontal plane applied as a “knife” to slice the volume, where the sample needs to be manually flattened to obtain more information [16], which can cause contamination or damage. However, imaging of non-planar surfaces (especially in very thin layers) such as tilted or curved samples obscures the target layer due to strong reflection from non-target layers, resulting in low image contrast.

Although a possible solution is to disregard strong reflection layers by selecting only target layers using “virtual flattening” via surface thresholding [6], its potential for curved en face imaging has been largely overlooked in general OCT applications. Existing works either are designed specifically for ophthalmology [17,18], require special lens design [19], or do not focus on visualizing en face images [20,21]. We have added a comparison of existing literature in Table A1.

In this study, we propose an arbitrary curved OCT en face extraction with adaptive threshold selection and data processing capability. Our algorithm adaptively selects appropriate thresholds, then calculates the shape of curved surface, while mitigating strong interferences caused by reflection from highly reflective samples. To verify the feasibility of this method, we applied our method to two challenging scenarios: (1) detecting thin latent fingerprints on a diffusive translucent tape and (2) measuring the ink thickness (by image frequency analysis) of a thermal-printed label—both of which are beyond the capabilities of traditional processing techniques due to low contrast within an image plane and the targets’ thin nature (as they are only a few microns thick). Since our method can also be adapted for other optical imaging techniques, and for rapid knowledge dissemination, we have also made our data and code publicly available.

## 2. Experimental Setup

### 2.1. SD-OCT System

**Figure 1:**
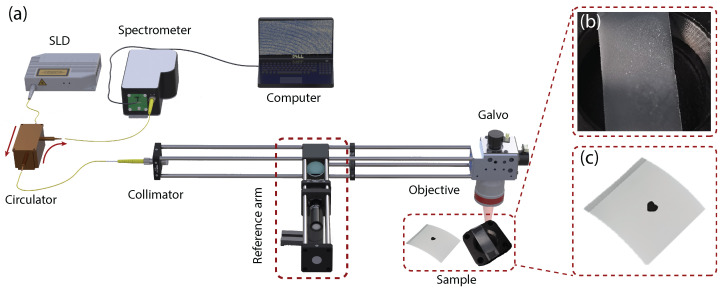
Experimental setup: (**a**) equipment and samples, (**b**) translucent tape with latent fingerprint, and (**c**) a thermal-printed label.

The spectral-domain optical coherence tomography (SD-OCT) system built in this study is shown in Figure 1a. The light source is a super luminescent light-emitting diode with a center wavelength of 850 nm, a bandwidth of 50 nm, and an output power of 15 mW. The axial resolution of the system is 6.4μm. The incident light is split into the sample arm and the reference arm by a beam splitter with a splitting ratio of 90:10 to enhance the signal from the samples. A dispersion compensator with the focusing lens is incorporated in the reference arm to minimize the dispersion mismatch between two arms. The interferogram formed from the backscattering of the sample arm and the back-reflecting of the reference arm is then recorded using a spectrometer with 0.05 nm spectral resolution.

### 2.2. OCT Volume

Three-dimensional OCT volume data consists of two lateral axes (named X and Y) and one axial axis (frequency). Our OCT volume contains 1024 voxels along the X-axis, 256 voxels along the Y-axis, and 2048 frequency-domain voxels along another othorgonal axis. To extract spatial information from this raw data, the source spectra are determined by calculating the median of each X-frequency-plane. Then a Fourier transform with the Hann window (to minimize spectral leakage) is applied to the source-subtracted frequency axis, resulting in 1024 voxels of spatial depth information (whereas the other half of the mirror image is discarded due to the limitation by Nyquist criterion in a real-valued signal). One can combine multiple scan volumes to improve the signal-to-noise ratio by a factor of square root of the scan count.

## 3. Curved Slicing Method

To automatically discover objects’ boundaries and layers, we extract foreground voxels (by Otsu-based binarization), detect the object’s structural morphology (by performing curve-fitting), and select the target layer (by weighing technique). The resulting 3D curve is then ready to be used for further analysis.

### 3.1. Foreground Voxels Extraction

**Figure 2:**
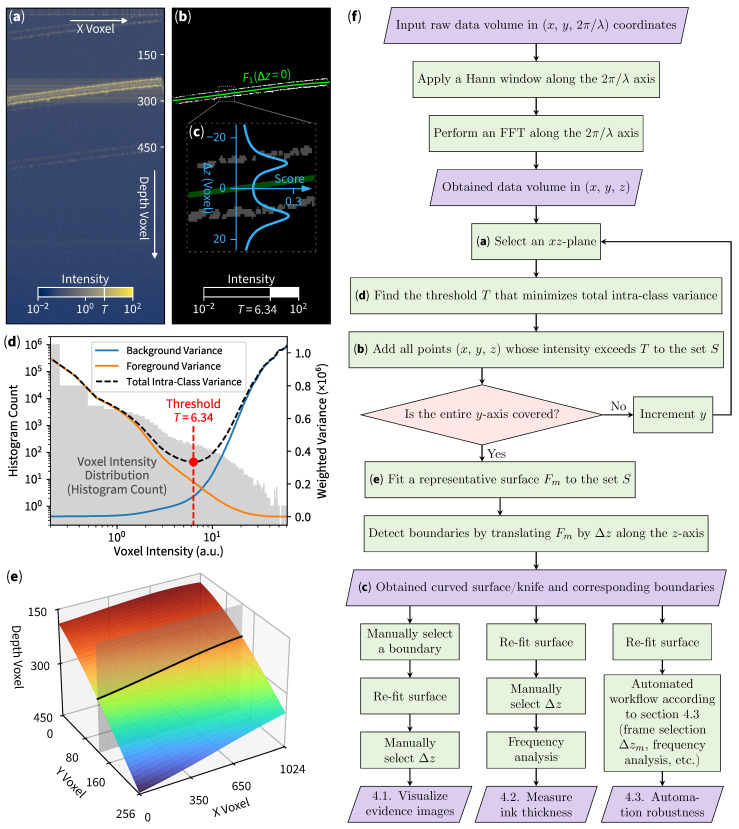
Curved slicing method. (**a**) Original B-scan (cross-sectional) slice with threshold indicated as *T*. (**b**) Image after binarization (white voxels) and surface-fitting $F_{1}\left(\Delta z=0\right)$ (green line). (**c**) Inset indicating boundary score for automated boundary detection. (**d**) Otsu’s thresholding operation, with slice voxel histogram in background (left axis) and variances calculations in foreground (right axis). (**e**) 3D surface indicating curved en face slice with the gray sheet as a cross-sectional B-scan used in (**a**,**b**). (**f**) Flow chart describing our method.

The binarization process separates the object of interest from the background, where Otsu’s method is utilized. Otsu’s technique automatically determines an optimal threshold value by maximizing the interclass variance between the background and foreground voxels. In simpler terms, if the total variance within each (foreground or background) group is minimized, the corresponding threshold is optimal. After a proper threshold is found, each voxel’s intensity in the original slice is compared to the calculated threshold. Voxels with intensities above the threshold, marked with *T* in Figure 2a, are classified as foreground (regions of interest), while those below are considered background. This process (illustrated in Figure 2d) creates a binary mask for each slice (Figure 2b).

### 3.2. Structural Morphology Detection

As the foreground voxels are identified through a binarization process, their locations are extracted to determine the object’s boundary. Curve fitting is performed on these 3D-voxel coordinates to best fit the boundary layers characterized by a surface equation (such as polynomial, spline, and others). If the surface is rough, one may use periodic functions or even use deep learning methods to model a relatively complicated surface. In our case, a polynomial surface equation will suffice:

(1) $$f\left(x,y\right)=\sum_{a=0}^{k}\sum_{b=0}^{k-a}C_{a,b}x^{a}y^{b}$$

with the polynomial degree k=3 (due to the nature of our sample) being used throughout this paper. To fit the curve, we select *N* representative voxels and find every $C_{a,b}$ that minimizes $\sum_{i}^{N}{\left[f\left(x_{i},y_{i}\right)-z_{i}\right]}^{2}$. We use Tikhonov regularization [22] to prevent overfitting by introducing a fix regularization variable α=0.1 for minimization of $||y-{Xw||}_{2}^{2}+{\alpha ||w||}_{2}^{2}$, according to the Scikit-learn library [23]. This step obtains a surface that best represents objects’ structural morphology while minimizing shot noises and rejecting reflection artifacts. We then describe the fitted surface as

(2) $$F_{m}\left(\Delta z\right)=f\left(x,y\right)+\Delta z$$

with unique label *m* to represent individual instances capable of shifting the depth axis up or down by Δz. We can then choose m=1 to represent the initial fitted curve (the green line in Figure 2b) as $F_{1}\left(0\right)$. The full 3D representation of the surface $F_{1}\left(0\right)$ illustrated in Figure 2e can be drawn by respectively iterating *x* and *y* over $x_{i}\in \left[0,1023\right]$ and $y_{i}\in \left[0,255\right]$.

### 3.3. Target Layer Selection

To automatically select a specific boundary layer, we can apply the weighing technique used for outlier rejection [24] to obtain

(3) $$\gamma \left(\Delta z\right)=\frac{1}{N}\sum_{i}^{N}\frac{1}{|f\left(x_{i},y_{i}\right)-z_{i}+\Delta z|+1}.$$

The key idea is that by shifting Δz along the depth axis *z*, the total distance between the points near the surface will decrease the denominator term, which consequently increases the sum of the inverse of distances. The addition of 1 is used to prevent each term from overflowing and is inherently used as a normalizing factor. From Figure 2c, when the green surface crosses each boundary, the boundary score (blue graph) will rise due to decreasing distances. Moreover, the magnitude of this score could be used as an approximation for inlier points selection. For example, if we would like to select the second (bottom) layer for further analysis, we first move the green surface down the depth axis to $F_{1}\left(\Delta z\approx 11\right)$, then the points that represents the second boundary will be the closest to the surface, which increases the boundary score (totalling around 30% of the volume foreground points). We then finally cut 70% of the points, classifying them as outliers, and repeat the curve-fitting process. The second curve-fitting process inherently eliminates noises from other layers due to outlier rejection.

## 4. Results and Discussions

We demonstrate the (1) capability of curved slicing by exploration of latent fingerprint (multi-layer surface extraction and visualization) and (2) extensibility by integrating additional 2D image frequency analysis for measuring the thickness of ink (image processing integrations).

### 4.1. Multi-Layer Surface Extraction and Visualization

**Figure 3:**
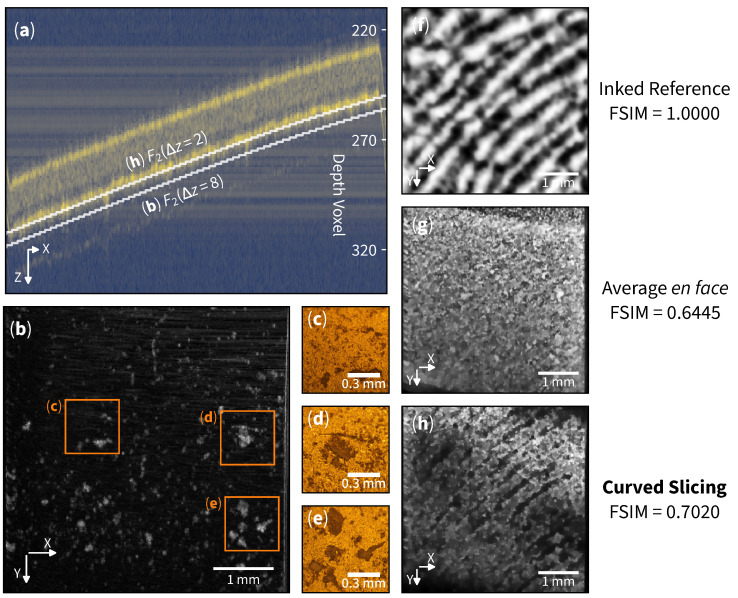
Latent fingerprint residue detection. (**a**) Cross-section of translucent tape with bright yellow lines indicating tape boundaries. $F_{2}\left(\Delta z\right)$ indicates the curved slicing function (selecting the second layer). (**b**) Curved en face slice at Δz=8 showing another layer of residue. (**c**–**e**) Microscopic images at each region. (**f**) Inked reference from fingerprint. (**g**) Traditional average en face from z=170 to z=435. (**h**) Curved en face slice at Δz=2 showing a layer of resolvable latent fingerprint pattern. Images (**b**,**f**–**h**) are enhanced for clarity.

Curved en face slicing emerges as a promising technique in forensic science, specifically in non-invasive exploration of latent fingerprint evidence. We have collected a fingerprint sample from a volunteer using the glue side of a translucent tape, followed by an OCT scan of the matte side, as depicted in Figure 1b. The resulting cross-section B-scan is shown in Figure 3a.

**Figure A1:**
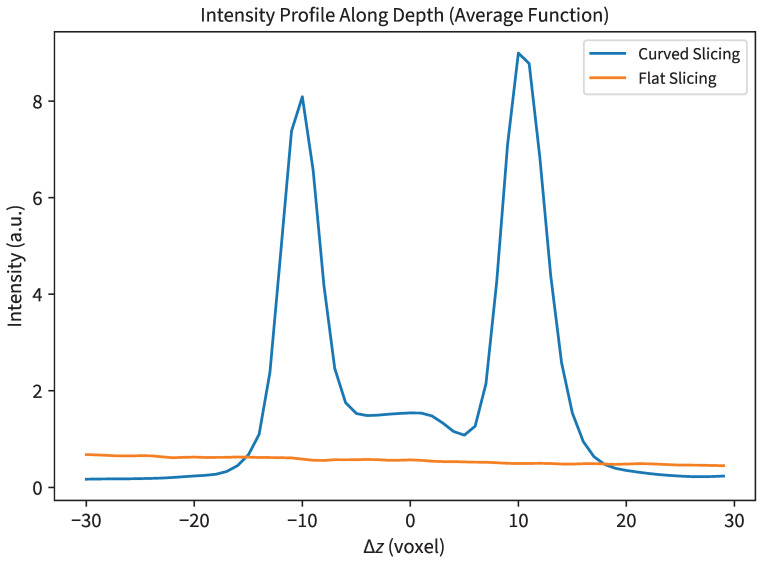
Average profile along the depth axis comparing curve slicing and flat slicing; sample according to multi-layer surface in Section 4.1.

The average en face image traditionally employed in biological sample analysis, as illustrated in Figure 3g, faces a significant challenge in visualizing sub-surface structures within the volume. A profile of curved versus flat slicing is also shown in the appendix (Figure A1). This limitation arises from the image (signal) being overshadowed by strong material reflections (interference). Unlike biological samples with complex structures, where each substructure typically exhibits high contrast (either high reflection or high absorption), the fingerprint information in this sample is confined to the superficial layers of the tape with relatively low contrast.

The flexibility of curved slicing around the glue layer of the tape allows us to explore specific layers in detail. We first define a curved slice on the second boundary as $F_{2}\left(\Delta z\right)$. Then, slicing on layer $F_{2}\left(\Delta z=2\right)$ reveals surface structures that exhibit latent fingerprint-like patterns. See Figure 3h. The Feature Similarity Index (FSIM) can be used to evaluate the structures’ similarity [25]; the curved slicing exhibits an FSIM of 0.7020 when compared to the inked reference Figure 3f.

Examining layer $F_{2}\left(\Delta z=8\right)$ uncovers dust-like residues scattered across the surface (see Figure 3b). The presence of these patterns exhibits a resemblance and is in good agreement with the microscopic images shown in Figure 3c–e. The evidence revealed by the curved slicing method can be crucial in forensic investigations, allowing detailed identification and analysis of foreign matter associated with a crime scene.

### 4.2. Image Processing Integrations

**Figure 4:**
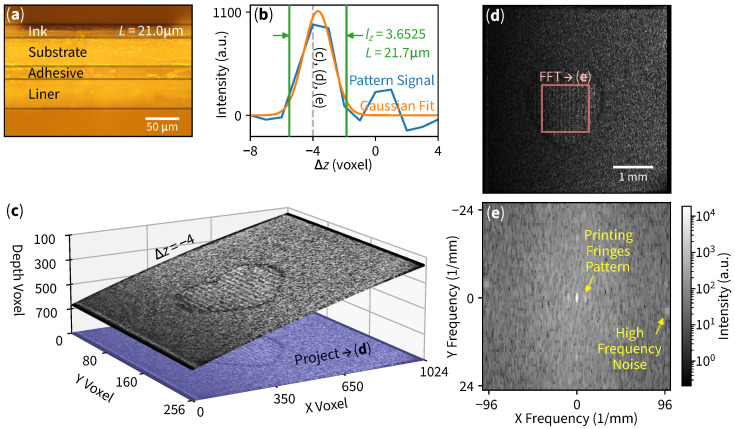
Ink thickness measurement in a thermal-printed label. (**a**) Cross-sectional microscopic image of a printed label paper showing ink thickness L=21.0μm. (**b**) Pattern signals at each Δz slice. The $1/e^{2}$ of the Gaussian curve fit amplitude determines the layer count $l_{z}$, which converts to ink thickness L=21.7μm. (**c**) Three-dimensional curved slice at Δz=−4 showing a heart pattern with its projection (or flattening). Images are enhanced for clarity. (**d**) Unprocessed en face at Δz=−4; the middle square region is used to perform 2D Fourier transform. (**e**) A two-dimensional frequency-domain image with DC in the center. Arrows indicate a periodic printing pattern and high frequency noise. The subtraction of intensities results in a data point of the pattern signal on (**b**).

The interoperability between our curved slicing method and digital signal processing techniques facilitates ink thickness measurement applications. Our objective is to effectively detect and count layers within the material’s structure. We used OCT to scan a thermal-printed label (surface-side), as indicated in Figure 1c, with its corresponding cross-sectional microscopic image Figure 4a.

The processing workflow begins with curved slicing at each layer, which in this example is at Δz=−4 (Figure 4c). The central region of the flattened curved slice reveals a periodic printing pattern in Figure 4d, which is then subjected to 2D FFT analysis as shown in Figure 4e.

The frequency-domain image reveals three bright low-spatial-frequency spots near the center: one representing the DC offset and two symmetric spots indicating fringes from the thermal printing process. Areas close to the edge of the image exhibit high-frequency behavior (speckle noise). Finally, we subtract the printing pattern spot with high-frequency noise to be a single “pattern signal” data point on Figure 4b on a blue line (with the current example slice denoted by a dashed line). By repeating the aforementioned workflow of slicing, projection, and Fourier transform for every layer, we are able to obtain the pattern signal.

The measurement process starts with the raw signal in Figure 4b. Subsequently, a Gaussian curve fitting is applied to this signal. A cutoff point (or signal width representative [26]) at $1/e^{2}$ of the Gaussian fit amplitude is considered for layer counting. To translate from voxel lengths to real-world lengths, we define physical domain coordinates as $X=M_{x}x$, $Y=M_{y}y$, and $Z=M_{z}z$ where $M_{x}$, $M_{y}$, $M_{z}$ are 4.8828, 19.5312, 4.2068 μm/voxel, respectively. Since the surface is tilted, the real thickness is proportional to the projected length of the gradient plane. The vector that defines a gradient plane at point $\left(x_{0},y_{0}\right)$ is defined by

(4) $$r={\left[\left(\frac{M_{z}}{M_{x}}\right)\frac{\partial f}{\partial x}\left(x_{0},y_{0}\right),\left(\frac{M_{z}}{M_{y}}\right)\frac{\partial f}{\partial y}\left(x_{0},y_{0}\right),1\right]}^{T}$$

with its corresponding unit vector $\hat{r}=r/\left|r\right|$. The layer thickness *L* is calculated by projecting layer count $l_{z}$ (in voxel) with basis $e_{z}$ onto vector $\hat{r}$, yielding

(5) $$L=n_{\mathrm{paper}}M_{z}l_{z}\left(e_{z}\cdot \hat{r}\right).$$

Substituting paper refractive index $n_{\mathrm{paper}}=1.557$ [27], $l_{z}=3.6525$ voxels and other parameters into (4) results in calculated thickness L=21.7μm. The thickness obtained from our curved slicing method is consistent with that of the cross-sectional microscopic image, demonstrating the high compatibility of our approach when integrated with other data processing techniques.

### 4.3. Automated Workflow Robustness Analysis

**Figure 5:**
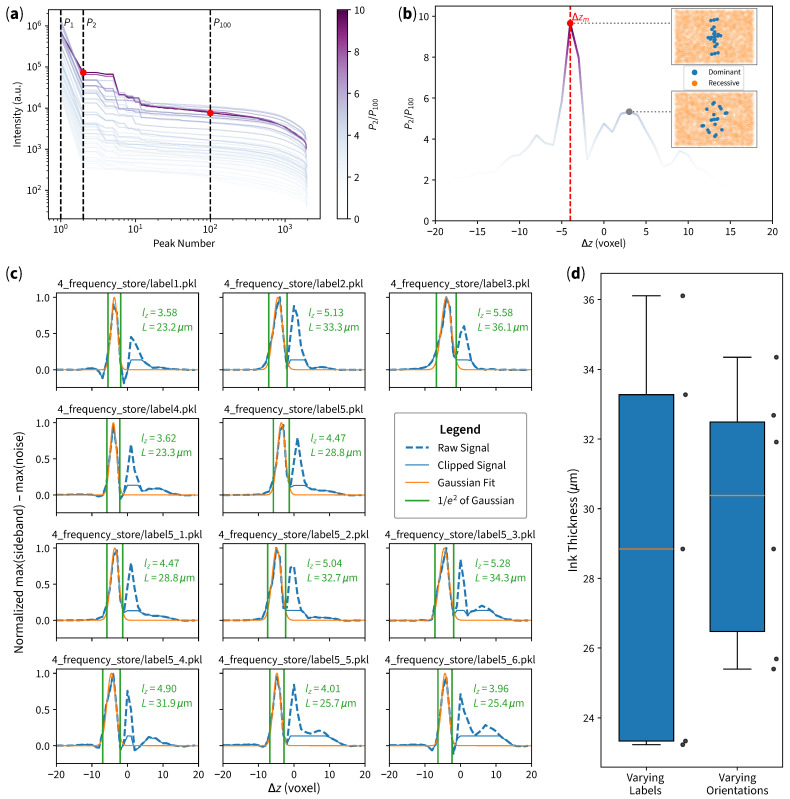
Automated ink thickness measurement results. (**a**) Intensity distribution sorted in descending order; each line represents each slice Δz, and the color of each line represents the ratio $P_{2}/P_{100}$ of each slice. (**b**) Ratio according to each Δz, where the maximum ratio corresponds to candidate slice $\Delta z_{m}$. The top inset shows dominant and recessive frequency-domain data points of $\Delta z_{m}$, where the points are aligned according to the inked pattern. The bottom inset shows frequency-domain data points of a non-candidate slice, where points are distributed in a random manner (with higher spatial frequency indicating possible noise). (**c**) Signal processing results of each case. (**d**) Box plot of measurement results, with standard deviations of 5.1802μm for different labels and 3.4322μm for different orientations.

To inspect the robustness of our curved slicing method, we have constructed an automated workflow of ink thickness measurement. We have printed five samples and selected a random sample to obtain OCT volumes in six different orientations. The experiment is conducted as follows:

1. Obtain the OCT volume.
2. Perform curved slicing to obtain the surface equation and its corresponding boundary (according to the flowchart in Figure 2f).
3. For each Δz∈[−20,19] slice, perform dimensionality reduction:
  3.1. Apply 2D FFT to obtain the 2D frequency domain.
  3.2. Reduce the dimension by applying maxpool to the frequency domain image.
  3.3. Extract locations and intensities in the frequency domain.
  3.4. Sort the information by intensity (as shown in Figure 5a, where each line represents each Δz slice).
4. Find the candidate slice $\Delta z_{m}$ which has the largest $P_{2}/P_{100}$ ratio, where $P_{2}$ is the second-largest intensity (probably the sideband, shown in Figure 5a as the middle dashed line), and $P_{100}$ is the 100th-largest intensity (probably noise, shown in Figure 5a as the right-most dashed line). The ratios of all slices are separately plotted as shown in Figure 5b for visualization purposes.
5. In the candidate slice $\Delta z_{m}$, divide frequency-domain data points into two sets: the 23 largest intensities as dominant locations and the rest as recessive locations (shown as the top inset in Figure 5b).
6. For each Δz:
  6.1. The largest intensity near dominant locations of $\Delta z_{m}$ is considered as the sideband.
  6.2. The largest intensity near recessive locations of $\Delta z_{m}$ is considered as noise.
  6.3. Signal is considered as sideband subtract noise. Refer to the blue dashed line plotted in Figure 5c.
7. Perform the initial Gaussian fit to the signal.
8. Clip the signal at the right side ($1/e^{2}$ of Gaussian) for curve-fitting stability. Refer to the blue solid line plotted in Figure 5c.
9. Perform the second Gaussian fit to the clipped signal. Refer to the orange line of plots in Figure 5c
10. Calculate the ink thickness according to (5).

The results are as shown in Figure 5, where panel (c) shows different measurement results (with a raw signal, clipped signal, and Gaussian fit), and panel (d) shows a box plot of both varying labels and varying orientations.

The candidate frame $\Delta z_{m}$ obtained by our workflow is very stable, achieving either $\Delta z_{m}=-4$ or $\Delta z_{m}=-5$. After the initial Gaussian optimization, signal clipping, and second Gaussian fit, the second Gaussian curve fitting achieves the peak of the curve at $l_{z}=-4.2\pm 0.7$ voxel, which is also stable, despite the changing samples and orientations. The consistency in peak locations and signal characteristics confirms the robustness of our method. The different labels achieved the standard deviation of 5.1802μm, while different orientations of one of the same labels achieved standard deviation of 3.4322μm, which indicates that the method is more sensitive to different labels rather than different orientations.

As evidenced by the insensitivity of samples and the varying orientations, our method is robust to different 3D curves samples that may exhibit different random noises. The surface re-fitting and candidate slice selection workflow ensures effective noise rejection as long as the curve can be explained as a surface equation. While some surface equations can be numerically unstable, we have not observed any overfitting tendency in our experiments. In fact, with a robust automated workflow, our technique can be extended to serve other imaging technologies that require 3D data processing, such as 3D X-ray and CT scans [28].

## 5. Conclusions

In this paper, curved en face slicing is a powerful technique for analyzing samples with complex surface structures. An adaptive threshold selection based on Otsu’s method is used for foreground selection, which serves as a basis for polynomial surface equation curve fitting (with Tikhonov regularization to prevent overfitting). A boundary score can also be used for automated boundary selection, where we can repeat the curve-fitting process for each boundary (which vary slightly). We can use the obtained “curved knife” to slice through each depth layer and extract en face images, in order to (1) reveal the latent fingerprint and its residues on different depths embedded under a translucent tape, (2) integrate data processing techniques (2D Fourier transform and Gaussian curve fitting) for ink thickness measurement of a thermal-printed label, and (3) automate the ink thickness measurement workflow. Future works may include complex surface modeling by means of generative adversarial networks for applications that require high precision in handling multilayer structures.

Our approach demonstrates versatility and potential for wide-ranging applications. For example, it can be used to detect counterfeit bank notes (with thickness measurement or pattern inspections), perform curved slicing to inspect surface damage in biological samples, and conduct quality inspections, all without destroying the evidence, specimens, or samples. With an automated workflow, curved slicing can be applied to any application that requires 3D volume processing to achieve meaningful results. As such, along with provided data and code, we envision numerous future research findings and practical applications.

## Data Availability

The original data and code presented in the study are openly available in FigShare at http://dx.doi.org/10.6084/m9.figshare.28645661, accessed on 3 May 2025.

## Abbreviations

The following abbreviations are used in this manuscript:

OCT	Optical coherence tomography.
SD-OCT	Spectral-domain optical coherence tomography.

## Appendix A

**Table A1:** Comparison of surface-flattening algorithms from existing studies.

Criteria	RTS ^a^	FMC ^b^	MAI ^c^	LED ^d^	HSL ^e^	This Work
Good contrast and sharpness of images	✓	✓	✓	✓	✓	✓
Designed for different samples		✓				✓
Discovery of multiple boundaries	✓				✓	✓
Slicing/flattening methodology	SW ^1^	SW ^1^	SW ^1^	SW ^1^	HW ^2^	SW ^1^
Focusing on obtaining en face images			✓			✓

^a^ Regularized thin-plate spline [20]. ^b^ Flat mirror calibration [21]. ^c^ Maximum A-scan intensity [17]. ^d^ Laplace edge detection [18]. ^e^ A hypercentric scanning lens with self-reference [19]. ^1^ Software-based flattening or slicing. ^2^ Hardware-based flattening or slicing.
